# Assessment of outcome and tolerability of high velocity nasal insufflation and continuous positive airway pressure in children with acute respiratory distress

**DOI:** 10.1186/s12887-026-06780-z

**Published:** 2026-04-11

**Authors:** Tarek A. Abdel Gawad, Hanan M. Ibrahim, Shrouk M. Awadallah, Mostafa H. Hassan, Sondos M. Magdy

**Affiliations:** 1 Department of Pediatrics, Faculty of Medicine, Ain Shams University, Cairo, Egypt; 2 Department of Radiology, Faculty of Medicine, Ain Shams University, Cairo, Egypt

**Keywords:** High-velocity nasal insufflation, Continuous positive airway pressure, Non-invasive ventilation, Pneumonia, Bronchiolitis, Lung ultrasound score

## Abstract

**Background:**

Acute respiratory distress remains a leading cause of mortality in children under five years of age. Non-invasive ventilation (NIV) can reduce the need for invasive mechanical ventilation. This study evaluated outcomes and tolerability of High-Velocity Nasal Insufflation (HVNI) versus Continuous Positive Airway Pressure (CPAP) in children with acute respiratory distress.

**Methods:**

This prospective cohort study included 80 children (1 month to 5 years) admitted to the PICU with acute respiratory distress after failure of low-flow nasal oxygen therapy. Patients received either HVNI (flow 1–2 L/kg/min; FiO₂ 21–100%) or CPAP (PEEP 5–12 cmH₂O; FiO₂ 21–100%). Patients were monitored for clinical response and readiness for weaning. Lung ultrasound was used to assess lung ultrasound score (LUS), and tolerability was assessed using the FLACC pain scale.

**Results:**

Pneumonia was the most common diagnosis in both groups (HVNI: 80%; CPAP: 82.5%, *p* = 0.78). Median NIV duration was comparable (HVNI: 5 [IQR 4–7] days vs. CPAP: 5 [IQR 4–6] days, *p* = 0.64), as was PICU length of stay (HVNI: 7 [IQR 6–10] days vs. CPAP: 7 [IQR 6–9] days, *p* = 0.71). There was no statistically significant difference in PICU outcomes or NIV failure rates between groups (*p* > 0.05). FLACC scores were lower in the HVNI group (median [IQR]: 2 [1–3] vs. 4 [3–5], *p* = 0.014), and sedation requirements were higher in the CPAP group (*p* = 0.016), indicating better tolerability with HVNI.

**Conclusion:**

HVNI and CPAP had comparable success rates, NIV failure rates, and PICU length of stay. CPAP was associated with better lung aeration, whereas HVNI was more comfortable and required less sedation. NIV modality should be individualized based on clinical condition and tolerance.

## Introduction

Acute respiratory failure is a medical emergency in infants and children; approximately 15–20% of affected children require intensive care and respiratory support [1], The World Health Organization and the American Academy of Pediatrics (AAP) both recommend oxygen supplementation at an arterial pulse oximetry (SpO2) lower than 90% because oxygen supply in children with acute lower respiratory tract infection is associated with reduced mortality [2].

Non-invasive ventilation is widely used in the respiratory management of severe cases of respiratory distress, high-flow nasal oxygen (HFNO) oxygen therapy provides oxygen-rich, heated, humidified gas to the patient’s nose at supraphysiologic flows sufficient to deliver a constant, precisely set high FiO2 [3, 4]. HFNO includes high flow nasal cannula (HFNC) and high velocity nasal insufflation (HVNI). HFNC flow rates reach up to 60 L/min, whereas HVNI delivers similar quantities of oxygen at flow rates up to 40 L/min due to increased velocity derived from a lower flow with a higher level of kinetic energy in the delivered gas. Both technologies facilitate rapid dead space flush [5], Exhalation is to the open air in all HFNO therapies. Mechanistically, HFNO provides high FiO2, reduces dead space, provides low levels of PEEP [6], and decreases breathing frequency and work of breathing [4].

The nasal continuous positive airway pressure is another management modality that combines supplemental oxygen with a positive end-expiratory pressure which decreases the inspiratory resistance, reduces atelectasis, reduces alveolar resistance, increases surface area of alveoli, and enhances ventilation and perfusion (V/Q) matching through PEEP [6], it has been shown to reduce the ventilation need duration and the overall hospitalization length in children with respiratory distress [7].

Lung ultrasound (LUS) has gained recognition as a straightforward, non-invasive, and real-time diagnostic tool for various respiratory conditions. Additionally, LUS can assist in guiding and evaluating the effectiveness of interventions targeting the lungs, such as fluid management, diuretic therapy, endotracheal intubation, ventilator adjustments, and chest tube insertion [8]. Clinicians can incorporate LUS into patient care to diagnose common pulmonary issues and enhance treatment outcomes, much like they rely on a stethoscope in routine practice [9].

Both high-flow nasal oxygen (HFNO) and continuous positive airway pressure (CPAP) are valuable modalities for managing pediatric patients with acute respiratory distress who do not respond to low-flow oxygen therapy. However, each technique has distinct advantages, disadvantages, and associated side effects. This study aimed to compare HVNI and CPAP in terms of tolerability, effectiveness, and side effects, utilizing various parameters, including lung ultrasonography, to evaluate their impact comprehensively.

To our knowledge, no previous pediatric study has simultaneously combined FLACC score (for patient comfort and tolerability), serial blood gas monitoring (for objective physiological assessment), and lung ultrasound (for dynamic imaging evaluation of lung aeration) to compare HVNI and CPAP in acute respiratory distress. Our study therefore addresses an important gap in the literature and has the potential to inform clinical decision-making in choosing the optimal modality for pediatric acute respiratory distress.

## Methods

This prospective cohort study included 80 children diagnosed with acute respiratory distress and admitted to the Pediatric Intensive Care Units (PICUs) at Ain Shams University Pediatric Hospital between April 2023 and March 2024. Eligible participants were aged between 1 month and 5 years of both sexes and required non-invasive respiratory support after failing to improve with low-flow nasal oxygen therapy. Failure of low-flow oxygen therapy was defined as the inability to maintain adequate oxygenation or ventilation with standard low-flow oxygen therapy as follows: Persistent SpO₂ < 92% on maximum tolerated flow of standard nasal cannula oxygen, Signs of increased work of breathing (tachypnea, nasal flaring, chest retractions, or use of accessory muscles), and/or Evidence of rising PaCO₂ on venous blood gas indicating inadequate ventilation necessitating escalation to NIV.

Patients were excluded if they met any of the following criteria:

1. Requirement for invasive mechanical ventilation due to respiratory failure at presentation.
2. Presence of multiple congenital anomalies or significant chronic comorbidities, including congenital heart disease, cardiomyopathy, neuromuscular disorders, chronic lung diseases (including conditions such as congenital diaphragmatic hernia with pulmonary hypoplasia, recurrent wheezing, skeletal deformities or other anomalies significantly affecting respiratory function), or a prior history of cardiopulmonary arrest.

Patients were enrolled consecutively and divided into two groups according to the type of respiratory support they received as part of standard clinical care:

- Group A (HVNI): Patients received high-flow nasal insufflation (HVNI) therapy using the Vapotherm 2000i device. The initial flow was set at 1–2 L/kg/min, and FiO₂ was titrated between 21% and 100% to maintain target oxygen saturations appropriate for age. Flow and FiO₂ were adjusted according to clinical response and gas exchange. Step-down from HVNI to standard low-flow oxygen was considered once patients demonstrated improved work of breathing, stable oxygenation (SpO₂ > 92% on FiO₂ ≤ 40%), and hemodynamic stability.
- Group B (CPAP): Patients received continuous positive airway pressure using the Medin SINDI device. The initial PEEP was set at 5 cmH₂O and increased up to 12 cmH₂O if needed, with FiO₂ titrated between 21% and 100% to maintain target oxygen saturations. CPAP was weaned by gradually reducing PEEP and FiO₂ once patients achieved stable oxygenation and reduced respiratory distress.
- Escalation criteria: Patients were escalated from one modality to another, or to invasive ventilation, if they demonstrated persistent hypoxemia, rising PaCO₂ with respiratory acidosis, worsening respiratory distress, or intolerance of the interface despite maximal settings.

Allocation to HVNI or CPAP was based on predefined unit practice and the treating physician’s assessment of respiratory distress severity, gas exchange abnormalities, and interface tolerance at presentation. Patients were not randomized, and allocation was not alternated. Study investigators did not influence treatment assignment. This non-randomized approach reflects real-world clinical practice but may introduce selection bias.

All participants underwent detailed history taking, including age, sex, cause of PICU admission, and past medical history to confirm adherence to exclusion criteria. Comprehensive clinical examinations were performed, covering the chest, cardiac, abdominal, and neurological systems. Patients were closely monitored for vital signs, including heart rate, blood pressure, respiratory rate, oxygen saturation, and temperature, as well as respiratory mechanics according to their respective ventilation group.

Respiratory distress severity was graded as follows:

- Grade 2 (moderate): tachypnea with nasal flaring and mild chest retractions, maintaining SpO₂ ≥ 92% on supplemental oxygen.
- Grade 3 (severe): marked tachypnea with severe chest retractions, use of accessory muscles, and SpO₂ < 92% despite oxygen therapy.
- Grade 4 (very severe): respiratory failure with cyanosis, altered consciousness, or signs of impending respiratory arrest.

Radiological evaluations included chest X-rays taken on admission and at weaning, alongside lung ultrasonography to assess lung ultrasound scores (LUS) at three time points: admission, 24 h after starting NIV, and at weaning. All LUS and CXR assessments were performed and interpreted by either by Dr. Shorouk Awadallah, Lecturer of Radiodiagnosis, or by Dr. Mostafa Hussain, who had received structured training under Dr. Awadallah prior to study initiation. This ensured consistency and reliability of the LUS assessments across all patients.

LUS was performed using 3–16 MHz High Frequency Linear Array Transducer using Samsung HM70 EVO Portable Ultrasound Machine, each intercostal space of upper and lower parts of the anterior, lateral and posterior regions of the left and right chest wall was carefully examined, making twelve lung regions. LUS scores ranged from 0 (normal aeration),1 Point Moderate loss of aeration (Multiple B-lines either regularly spaced or irregularly spaced), 2 Points Severe loss of aeration (Multiple coalescent B-lines) to 3 (complete aeration loss) for each lung, with total scores between 0 and 18 in each lung.

All laboratory investigations were performed at Ain Shams University Hospitals according to standard operating procedures and the manufacturers’ instructions.

Complete Blood Count (CBC): Hemoglobin (Hb), platelets (PLTs), and white blood cell (WBC) counts were analyzed using the XN-1000 analyzer (Sysmex Corporation, Kobe, Japan).

C-reactive Protein (CRP): levels were measured by the immunoturbidimetric method using the Roche Cobas c501, with reagent kits provided by the manufacturer.

Blood Gas Analysis: the measurements were conducted using the ABL800 FLEX blood gas analyzer (Radiometer Medical, Brønshøj, Denmark).

Blood gases were obtained at baseline (prior to support initiation), at 1–2 h after initiation, and then every 6–8 h during the first 24 h, or sooner if clinical deterioration occurred, Blood gases were also repeated at the time of escalation or transition between modalities.

Blood Culture: Blood cultures were performed using the BACT/ALERT^®^ 3D microbial detection system (bioMérieux, Marcy-l’Étoile, France). Positive culture bottles were subcultured onto blood agar, MacConkey agar, and chocolate agar, followed by bacterial identification using standard biochemical tests and confirmed by the VITEK^®^ 2 Compact system (bioMérieux).

Sputum Culture: Sputum samples were cultured on blood agar, chocolate agar, and MacConkey agar, incubated at 37 °C for 24–48 hours. Bacterial identification was based on colony morphology, Gram staining, and standard biochemical tests.

Respiratory Viral Panel: Respiratory viral pathogens were detected using the BIOFIRE^®^ Respiratory Panel 2.1 (RP2.1, BioFire Diagnostics, Salt Lake City, UT, USA), a multiplex PCR-based assay detecting 22 viral and bacterial pathogens from nasopharyngeal swabs. The assay was conducted following the manufacturer’s protocol.

### Assessment of tolerability and NIV failure

In this study, tolerability was defined as the child’s ability to continue the assigned mode of non-invasive ventilation without requiring discontinuation due to discomfort or behavioural distress. Tolerability was assessed using the FLACC score [10], which evaluates facial expression, leg movement, activity, cry, and consolability, where Sedation was standardized and guided by FLACC score categories:

Score 0: no sedation, Score 1–3: non-medical interventions (e.g., pacifiers or screen time), Score 4–10: dexmedetomidine infusion dose (0.2–0.7 µg/kg/hr), Escalation from non-pharmacologic to pharmacologic sedation was predefined if agitation persisted.

Dexmedetomidine was selected as the exclusive sedative agent due to its favourable safety profile, offering sedation and anxiolysis without significant respiratory compromise, which makes it more suitable than alternative agents for children on non-invasive ventilation.

FLACC scores were recorded at baseline before initiation of support, at 1 hour after initiation, and subsequently every 4 hours during the first 24 hours, and then once per nursing shift, or sooner if clinical deterioration occurred.

NIV failure was defined as persistent or worsening respiratory distress, hypoxemia, or hypercapnia despite maximal NIV support, or the inability to tolerate the interface, necessitating escalation to invasive mechanical ventilation. For patients who transitioned to another mode of respiratory support, the reasons were recorded and categorized as clinical deterioration, procedural/medical requirement for intubation, or intolerance/agitation with the initial support. These criteria allowed us to objectively distinguish between treatment failure due to disease progression and discontinuation due to poor tolerability.

### Statistical analysis

Recorded data were analyzed using the statistical package for social sciences, version 26.0 (SPSS Inc., Chicago, Illinois, USA). Quantitative variables were presented as mean ± standard deviation for parametric data or median with interquartile range for non-parametric data. Qualitative variables were presented as numbers and percentages. Normality was assessed using the Kolmogorov-Smirnov and Shapiro-Wilk tests. Statistical significance was determined using:

- Independent-samples t-test for comparing two means (parametric data).
- Mann-Whitney U test for two-group comparisons (non-parametric data).
- Chi-square test for qualitative data comparisons.

### Probability (*P*-value)

A two-sided p-value < 0.05 was considered statistically significant.

#### Sample size calculation

Based on the findings of Xu et al. (2021), who reported a mean ICU stay of 7.2 ± 2.4 days in one group versus 9.5 ± 3.1 days in another, the required sample size to detect a statistically significant difference with an alpha error of 5% and 80% power was estimated to be 80 patients (40 in each group). Sample size was calculated using STATA version 10.

## Results

This prospective cohort study included 80 children (47 males, 59%) aged 1 month to 5 years who were admitted to the PICU between April 2023 and March 2024 with acute respiratory distress after failure of low-flow nasal oxygen therapy. Diagnoses were pneumonia (81%), bronchiolitis (15%), and complicated pneumonia (4%), with no significant differences between groups at baseline (Table 1).

**Table 1:** Patients characteristics and initial presentation

		Group A (HVNI)	Group B (CPAP)	Test value	*P*-value	Sig.
		No. = 40	No. = 40			
Age (month)	Median (IQR)	4 (2–13)	3 (1.75–5.5)	−1.558≠	0.119	NS
	Range	1–48	1–42			
Gender	Male	23 (57.5%)	24 (60.0%)	0.052*	0.820	NS
	Female	17 (42.5%)	16 (40.0%)			
Date of admission (month)	April 2023	1 (2.5%)	1(2.5%)	10.269*	0.215	NS
	May 2023	1 (2.5%)	0 (0.0%)			
	June 2023	1 (2.5%)	1 (2.5%)			
	July 2023	1 (2.5%)	1 (2.5%)			
	August 2023	1 (2.5%)	0 (0.0%)			
	Sep. 2023	4 (10.0%)	0 (0.0%)			
	Oct. 2023	3 (7.5%)	3 (7.5%)			
	Nov. 2023	4 (10.0%)	2 (5.0%)			
	Dec. 2023 Jan 2024 Feb 2024 March 2024	11 (27.5%) 10 (25%) 2 (5.0%) 1 (2.5%)	13 (32.5%) 11 (27.5%) 7 (17.5%) 1(2.5%)			
Diagnosis	Bronchiolitis	6 (15.0%)	6 (15.0%)	3.138*	0.208	NS
	Pneumonia	31 (77.5%)	34 (85.0%)			
	Complicated pneumonia	3 (7.5%)	0 (0.0%)			
RR (breath/min)	Mean ± SD	45.22 ± 10.75	48.72 ± 9.3	−1.558•	0.123	NS
	Range	25–60	27–63			
HR (beat/min)	Mean ± SD	147.58 ± 13.82	151.37 ± 14.25	−1.211•	0.230	NS
	Range	120–173	118–170			
Temp ©	Mean ± SD	37.95 ± 0.85	37.9 ± 0.75	0.265•	0.792	NS
	Range	36.1–39.1	36.2–39.2			
Fever	No	14 (35.0%)	14 (35.0%)	0.000*	1.000	NS
	Yes	26 (65.0%)	26 (65.0%)			
Cyanosis	No	34 (85.0%)	35 (87.5%)	0.105*	0.745	NS
	Yes	6 (15.0%)	5 (12.5%)			
GIT symptoms	No	36 (90.0%)	39 (97.5%)	1.920*	0.166	NS
	Yes	4 (10.0%)	1 (2.5%)			
Respiratory distress before NIV	Grade 2	11 (27.5%)	13 (32.5%)	0.280*	0.869	NS
	Grade 3	23 (57.5%)	22 (55.0%)			
	Grade 4	6 (15.0%)	5 (12.5%)			

^*^Chi-square test, ^≠^Mann-Whitney test, ^•^Independent t-test

At presentation, all patients had cough and 65% had fever. Respiratory distress severity was grade 2 in 30%, grade 3 in 56.2%, and grade 4 in 13.8%, with no significant between-group differences (Table 1).

Baseline laboratory findings and microbiological results were comparable between groups and are summarized in Table 2. Respiratory PCR testing was performed in 57 children; RSV was the most frequently detected virus. Blood and sputum cultures were positive in a minority of cases (Table 2).

**Table 2:** Initial laboratory workup for both groups

		Group A (HVNI)	Group B (CPAP)	Test value	P-value	Sig.
		No. = 40	No. = 40			
Hemoglobin(g/dL)	Mean ± SD	10.33 ± 1.27	10.27 ± 0.98	0.227•	0.821	NS
	Range	7.2–13.6	8–13			
	Normal	18 (45.0%)	24 (60.0%)	1.805*	0.179	NS
	Low	22 (55.0%)	16 (40.0%)			
	High	0 (0.0%)	0 (0.0%)			
Platelet (10^3^/UL)	Median (IQR)	455.5 (340.5–599.5)	480.5 (384.5–577.5)	−0.351≠	0.725	NS
	Range	149–895	112–1062			
	Normal	16 (40.0%)	10 (25.0%)	2.208*	0.332	NS
	Low	1 (2.5%)	2 (5.0%)			
	High	23 (57.5%)	28 (70.0%)			
Neutrophil (10^3^/UL)	Median (IQR)	5.2(3.2–7.55)	3.75(2.75–6.5)	−1.449≠	0.147	NS
	Range	1.4–29	1.7–18			
	Normal	23 (57.5%)	30 (75.0%)	2.739*	0.098	NS
	Low	0 (0.0%)	0 (0.0%)			
	High	17 (42.5%)	10 (25.0%)			
Lymphocytes (10^3^/UL)	Median (IQR)	3.7(2.2–5.5)	4.45(2.35–6)	−0.443≠	0.658	NS
	Range	1–12	0.8–12.6			
	Normal	19 (47.5%)	20 (50.0%)	2.270*	0.321	NS
	Low	20 (50.0%)	16 (40.0%)			
	High	1 (2.5%)	4 (10.0%)			
C- reactive protein (mg/L)	Median (IQR)	17.25(1.8–42)	9.45(3.35–31)	−0.501≠	0.617	NS
	Range	0.4–344	0–191			
	Negative	16 (40.0%)	17 (42.5%)	0.052*	0.82	NS
	Positive	24 (60.0%)	23 (57.5%)			
BUN (mg/dL)	Median (IQR)	10(8.5–12)	10(7.5–11.5)	−1.266≠	0.205	NS
	Range	4–41	5–20			
	Normal	38 (95.0%)	39 (97.5%)	0.346*	0.556	NS
	High	2 (5.0%)	1 (2.5%)			
Creatinine (mg/dL)	Mean ± SD	0.37 ± 0.13	0.32 ± 0.11	1.774•	0.08	NS
	Range	0.2–0.8	0.2–0.8			
	Normal	37 (92.5%)	38 (95.0%)	0.347*	0.841	NS
	High	2 (5.0%)	1 (2.5%)			
ALT (U/L)	Median (IQR)	14.5(12–23.5)	13.5(12–19.5)	−0.029≠	0.977	NS
	Range	6–49	8–112			
	Normal	40 (100.0%)	38 (95.0%)	2.051*	0.152	NS
	High	0 (0.0%)	2 (5.0%)			
Serum albumin (g/dL)	Mean ± SD	3.81 ± 0.4	3.81 ± 0.43	0.000•	1.000	NS
	Range	3–4.6	2.9–4.8			
	Normal	32 (80.0%)	34 (85.0%)	0.346*	0.556	NS
	Low	8 (20.0%)	6 (15.0%)			
Initial pH	Mean ± SD	7.34 ± 0.06	7.36 ± 0.06	−1.675•	0.098	NS
	Range	7.23–7.52	7.26–7.55			
Initial PaCO2	Mean ± SD	41.98 ± 10.22	45.48 ± 9.93	−1.554•	0.124	NS
	Range	26–60	29–68			
Initial HCO3	Mean ± SD	21.56 ± 2.58	22.26 ± 2.06	−1.347•	0.182	NS
	Range	16–26	18–27.5			
Respiratory panel PCR multiplex#	Not done	10 (25%)	*13 (32.5%)*	1.818*	*0.178*	*NS*
	No organism	1 (3.3%)	*1 (3.7%)*	0.000*	*1.000*	*NS*
	RSV	15 (50%)	*15 (55.6%)*	0.000*	*1.000*	*NS*
	Influenza	3 (10%)	*5 (18.5%)*	0.556*	*0.456*	*NS*
	Covid-19	1 (3.3%)	*1 (3.7%)*	0.000*	*1.000*	*NS*
	Adenovirus	1 (3.3%)	*1 (3.7%)*	0.000*	*1.000*	*NS*
	Parainfluenza	3 (10%)	*0 (0.0%)*	3.117*	*0.077*	*NS*
	Rhino virus	8 (26.7%)	*3 (11.1%)*	2.635*	*0.105*	*NS*
	Step. pneumoniae	2 (6.7%)	*1 (3.7%)*	0.346*	*0.556*	*NS*
Blood cultures#	Not done	3 (7.5%)	4 (10.0%)	5.743*	0.332	NS
	No growth	27/37 (73%)	33/36 (91.7%)			
	Staphylococci (CoNS)	4/37 (10.8%)	2/36 (5.6%)			
	Staph epidermidis	2/37 (5.4%)	1/36 (2.8%)			
	S. hominis	3/37 (8.1%)	0 (0.0%)			
	S. aureus	1/37 (2.7%)	0 (0.0%)			
Sputum cultures#	Not done	10(25.0%)	10(25.0%)	2.888*	0.717	NS
	No growth	24/30 (80.0%)	23/30 (76.7%)			
	Strep. pneumoniae	4/30 (13.3%)	2/30 (6.7%)			
	MRSA	0(0.0%)	1/30 (3.3%)			
	Klebsiella pneumoniae	2/30 (6.7%)	3/30 (10%)			
	Enterobacter species	0(0.0%)	1/30 (3.3%)			

*HVNI* High velocity Nasal Insufflation, *CPAP* Continuous Positive Airway Pressure, *RSV* Respiratory syncytial virus, *Strep pneumoniae* Streptococcus pneumoniae, *CoNS* Coagulase-negative staphylococci, *S. hominis* Staphylococcus hominis, *S. aureus* Staphylococcus aureus, *S. epidermidis* Staphylococcus epidermidis, *MRSA* Methicillin-resistant Staphylococcus aureus, *pH* Potential hydrogen, *PaCo2* Partial pressure of carbon dioxide (mmHg), *HCO3* Bicarbonate

*P*-value < 0.05: Significant; *P*-value < 0.01: Highly significant

^*^Chi-square test, ^≠^Mann-Whitney test, ^•^Independent t-test # Percentages for microbiological investigations (PCR, blood culture, and sputum culture) were calculated based on the number of patients in whom the test was performed, excluding cases labelled as “not done”.

Baseline chest radiograph patterns are presented in Table 2. Baseline lung ultrasound assessment showed comparable initial LUS values between groups (Table 3). Over time, LUS improved in both groups; however, follow-up and pre-weaning LUS scores were significantly lower in the CPAP group for both lungs (Table 3), indicating greater improvement in lung aeration.

**Table 3:** Comparison between HVNI and CPAP groups

		(HVNI) Group	(CPAP) Group	Test value	*P*-value	Sig.
		No. = 40	No. = 40			
Flow (L/kg)	Mean ± SD	1.8 ± 0.37	–	–	–	–
	Range	1–2	–			
Peep (cmH2o)	Mean ± SD	–	5.63 ± 1	–	–	–
	Range	–	5–8			
Fio2 (%)	Mean ± SD	48.38 ± 13.89	39.65 ± 9.98	3.228•	0.002	HS
	Range	30–100	21–70			
Follow up blood Gaes (1–2 h after NIV initiation)						
pH follow-up	Mean ± SD	7.35 ± 0.08	7.39 ± 0.03	−3.159•	0.002	HS
	Range	7.2–7.48	7.33–7.47			
PaCO2 (mmHg) follow-up	Mean ± SD	44.5 ± 8.1	39.55 ± 5.35	3.226•	0.002	HS
	Range	34 − 60	28 − 60			
HCO3(mmol/L) follow-up	Mean ± SD	22.93 ± 2.03	22.96 ± 1.93	−0.073•	0.942	NS
	Range	20–29	19.3–29			
Difference (delta change) in blood gases parameters^+^						
pH	Mean ± SD	0.01 ± 0.10	0.03 ± 0.07	−0.251≠	0.802	NS
PaCO2 (mmHg)	Mean ± SD	2.53 ± 8.31	−5.93 ± 8.86	−4.103≠	0.000	HS
HCO3(mmol/L)	Mean ± SD	1.37 ± 2.81	0.7 ± 2.97	−1.154≠	0.248	NS
Lung ultrasound score						
1 st RT LUS	Median (IQR)	4(2–6)	4(4–5)	−0.352≠	0.725	NS
	Range	2–10	0–6			
Follow-up RT LUS	Median (IQR)	4 (3–5)	3 (2–3)	−3.182≠	0.001	HS
	Range	0–10	0–6			
Last RT LUS	Median (IQR)	3 (1–4)	2 (1–2)	−3.682≠	0.000	HS
	Range	0–8	0–3			
1 st LT LUS	Median (IQR)	3(2–4.5)	3(2–4)	−1.089≠	0.276	NS
	Range	1–6	0–6			
Follow-up LT LUS	Median (IQR)	3 (2–4)	2 (1–3)	−2.980≠	0.003	HS
	Range	0–8	0–6			
Last LT LUS	Median (IQR)	2(1–3)	1(0–2)	−2.654≠	0.008	HS
	Range	0–8	0–4			
Tolerance and sedation						
FLACC Score	Median (IQR)	3(2–4)	4(3–5)	−2.464≠	0.014	S
	Range	2 − 6	1 − 6			
Sedation	No	25(62.5%)	13(32.5%)	8.289*	0.016	S
	Dexmedetomidine	10(25.0%)	22(55.0%)			
	Non-medical Treatment (Pacifier, Screen)	5(12.5%)	5(12.5%)			
Outcome						
Complications	Sepsis	2 (5.0%)	3 (7.5%)	0.213*	0.644	NS
	Carditis	1 (2.5%)	1 (2.5%)	0.000*	1.000	NS
	Pneumothorax	2 (5.0%)	0 (0.0%)	2.051*	0.152	NS
NIV stay (Days)	Median (IQR)	5(3–7)	4(3.5–5.5)	−0.638≠	0.523	NS
	Range	2–15	2–30^#^			
Length of stay in PICU (Days)	Median (IQR)	7 (6–10)	7 (6–8)	−0.486≠	0.627	NS
	Range	4–18	4–33			
Failure	Improved	35 (87.5%)	32 (80.0%)	1.134*	0.567	NS
	Shift to another NIV mode	3 (7.5%)	6 (15.0%)			
	Shift to invasive ventilation	2 (5.0%)	2 (5.0%)			
Death	No	38 (95.0%)	38 (95.0%)	0.000*	1.000	NS
	Yes	2 (5.0%)	2 (5.0%)			

*Fio2* Fraction of inspired Oxygen (%), *pH* Potential hydrogen, *PaCo2* Partial pressure of carbon dioxide (mmHg), *HCO3* Bicarbonate, *LUS* lung ultrasound score

+Delta change = follow-up value minus baseline value, where baseline blood gases were obtained before NIV initiation and follow-up at 1–2 h after initiation

^#^The maximum NIV duration in the CPAP group reflects cumulative use in a single patient with complicated pneumonia, pneumothorax, and sepsis

*P*-value < 0.05: Significant; *P*-value < 0.01: Highly significant

^*^Chi-square test, ^≠^Mann-Whitney test, ^•^Independent t-test

Ventilatory parameters differed between groups: the HVNI group received a mean flow of 1.8 ± 0.37 L/kg/min, whereas the CPAP group received a mean PEEP of 5.63 ± 1 cmH₂O (range 5–8). FiO₂ requirements were lower in the CPAP group (39.65 ± 9.98% vs. 48.38 ± 13.89%, *p* = 0.002). Follow-up blood gases obtained 1–2 hours after NIV initiation showed a greater reduction in PaCO₂ in the CPAP group (delta PaCO₂: −5.93 ± 8.86 vs. 2.53 ± 8.31 mmHg, *p* < 0.001) and a higher follow-up pH (Table 3).

Tolerability was better with HVNI. FLACC scores were lower in the HVNI group (median [IQR] 3 [2–4] vs. 4 [3–5], *p* = 0.014), and sedation was required more frequently in the CPAP group (*p* = 0.016) (Table 3).

Overall, 76 children (95%) were discharged from the PICU and 4 (5%) died after escalation to invasive ventilation. Complications included sepsis (*n* = 5), carditis (*n* = 2), and pneumothorax (*n* = 2), with no significant differences between groups (Table 3). There were no statistically significant between-group differences in NIV duration, NIV failure categories, PICU length of stay, or mortality (Table 3). One child in the CPAP group required a prolonged cumulative NIV duration (30 days) due to recurrent respiratory deterioration with pneumothorax and sepsis (Table 3).

## Discussion

In this prospective cohort study of 80 children with acute respiratory distress after failure of low-flow nasal oxygen therapy, both HVNI and CPAP achieved high overall success rates with no significant differences in NIV failure rates, NIV duration, PICU length of stay, or mortality. CPAP was associated with better early gas exchange (greater PaCO₂ reduction) and greater improvement in lung aeration on lung ultrasound, whereas HVNI was better tolerated, with lower FLACC scores and reduced need for sedation.

Our findings align with prior pediatric studies [11, 12] comparing CPAP and high-flow respiratory support, in which CPAP was more effective in improving ventilation and lung recruitment, while high-flow modalities were generally more comfortable and easier to tolerate. In our cohort, CPAP was associated with significantly improved PaCO₂ clearance and greater LUS improvement over time, supporting its physiological advantage in lung recruitment. Conversely, HVNI was associated with better comfort and lower sedation requirements, consistent with evidence [13] that interface tolerance is a key determinant of NIV continuation in young children.

Lung ultrasound proved valuable for monitoring response to non-invasive respiratory support. Serial LUS assessment demonstrated improvement in both groups, with a significantly greater reduction in follow-up and pre-weaning LUS scores in the CPAP group. These findings support incorporating bedside LUS into clinical monitoring of pediatric respiratory distress, especially when evaluating lung recruitment and readiness for weaning, in line with previous studies demonstrating its reliability as a non-invasive bedside tool [14].

### Clinical implications

These results highlight the complementary roles of CPAP and HVNI. CPAP may be preferred when hypercapnia or lung recruitment is a priority, whereas HVNI may be advantageous when comfort, interface tolerance, and reduced sedation are important. Individualized selection based on clinical status and tolerance may optimize outcomes and reduce escalation. The effectiveness of HVNI in mild to moderate respiratory failure may be partly explained by its ability to improve oxygenation and reduce respiratory distress through end-expiratory CO₂ washout [15].

### Complications and safety

Complications were infrequent and similar between groups, consistent with previous reports [16, 17] . Pneumothorax and sepsis were uncommon and occurred primarily in children with more severe disease. Careful interface selection, monitoring, and standardized escalation criteria remain essential to minimize adverse events during NIV. Nasal trauma remains a recognized complication of CPAP, with reported rates varying depending on the interface used [18, 19].

### Limitation of the study

This study has several limitations that should be acknowledged. First, it was conducted in a single center, which may limit the generalizability of the findings to other settings. Second, patient allocation to HVNI or CPAP was non-randomized and based on physician decision, introducing the potential for selection bias, although uniform eligibility criteria and standardized outcome measures were applied to mitigate this risk. Third, tolerability was assessed using the FLACC score, which, while simple, validated, and practical for repeated bedside use, does not comprehensively evaluate sedation or agitation compared to scales such as COMFORT-B or SBS. This may have reduced the granularity of tolerability assessment. Fourth, FLACC scoring was performed by bedside nurses aware of the treatment modality, introducing possible observer bias, although standardized training and predefined assessment intervals were used to minimize variability. Fifth, we did not quantify the effective PEEP generated by HVNI, which could have provided a more direct physiological comparison with CPAP. Finally, some analyses were retrospective in nature, which carries an inherent risk of bias.

## Conclusion

Our study demonstrated that both CPAP and HVNI are effective in managing pediatric acute respiratory distress. CPAP was associated with a greater reduction in lung ultrasound scores over time and improved PaCO₂ clearance, while HVNI was better tolerated, as reflected by lower FLACC scores. Importantly, there were no significant differences between the two groups in terms of PICU length of stay, NIV duration, or intubation rates. These findings support the role of both modalities in the pediatric PICU, with the choice of support tailored to patient-specific clinical condition and tolerance.

## Data Availability

The datasets used and/or analyzed during the current study are available from the corresponding author on reasonable request.

## Abbreviations

HVNI: High flow nasal insufflation
CPAP: Continuous positive airway pressure
PEEP: Positive end expiratory pressure
NIV: Non-invasive ventilation
HFNO: High flow nasal oxygen
HFNC: High flow nasal cannula
PICU: Pediatric intensive care unit
LUS: Lung ultrasound score
LOS: Length of stay
CRP: C- reactive protein
WHO: World of health organization
AAP: American academy of pediatrics
